# Outcomes of surgical resection and intraoperative electron radiotherapy for patients with para-aortic recurrences of gastrointestinal and gynecologic malignancies

**DOI:** 10.1186/s13014-023-02289-2

**Published:** 2023-06-02

**Authors:** Jacob Hall, Jessica Wilson, John Shumway, Ted K. Yanagihara, Joel Tepper, Benjamin Calvo, Andrew Z. Wang, Kevin Pearlstein, Kyle Wang, Hong Jin Kim

**Affiliations:** 1 Department of Radiation Oncology, University of North Carolina School of Medicine, 101 Manning Drive CB #7512, Chapel Hill, NC 27514 USA; 2 Department of Radiation Oncology, Mayo Clinic, Rochester, MN USA; 3 Department of Surgical Oncology, Roswell Park Cancer Institute, Buffalo, NY USA; 4 Department of Radiation Oncology, University of Texas Southwestern Medical Center, Dallas, TX USA; 5 Department of Radiation Oncology, University of Cincinnati, Cincinnati, OH USA; 6 Division of Surgical Oncology, Department of Surgery, University of North Carolina, Chapel Hill, NC USA

**Keywords:** Intraoperative radiotherapy, Para-aortic recurrence, Colorectal metastases

## Abstract

**Background:**

Para-aortic lymph node (PALN) metastases from primary pelvic malignancies are often treated with resection, but recurrence is common. We report toxicity and oncologic outcomes for patients with PALN metastases from gastrointestinal and gynecologic malignancies treated with resection and intraoperative electron radiotherapy (IORT).

**Methods:**

We retrospectively identified patients with recurrent PALN metastases who underwent resection with IORT. All patients were included in the local recurrence (LR) and toxicity analyses. Only patients with primary colorectal tumors were included in the survival analysis.

**Results:**

There were 26 patients with a median follow up of 10.4 months. The rate of para-aortic local control (LC) was 77% (20/26 patients) and the rate of any cancer recurrence was 58% (15/26 patients). Median time from surgery and IORT to any recurrence was 7 months. The LR rate for those with positive/close margins was 58% (7/12 patients) versus 7% (1/14 patients) for those with negative margins (p = 0.009). 15% (4/26 patients) developed surgical wound and/or infectious complications, 8% (2/26 patients) developed lower extremity edema, 8% (2/26 patients) experienced diarrhea, and 19% (5/26 patients) developed an acute kidney injury. There were no reported nerve injuries, bowel perforations, or bowel obstructions. For patients with primary colorectal tumors (n = 19), the median survival (OS) was 23 months.

**Conclusions:**

We report favorable LC and acceptable toxicity for patients receiving surgical resection and IORT for a population that has historically poor outcomes. Our data show disease control rates similar to literature comparisons for patients with strong risk factors for LR, such as positive/close margins.

## Introduction/Background

Intraoperative electron radiotherapy (IORT) has been employed intermittently for over 100 years [1]. Interest in this modality has increased over the last few decades and there are approximately 90 centers world-wide with active IORT programs [2]. The rationale for IORT is rooted in improving the therapeutic ratio by physically excluding sensitive normal structures from irradiation and allowing for dose escalation in many cases. The modality has been used to treat primary tumors of the head and neck, central nervous system, thorax, abdomen, pelvis, and breast [3]. An area of utility is in select patients with intra-abdominal gross residual disease following surgical excision or positive margins. It can be difficult to prescribe adequate doses to intra-abdominal target volumes while achieving normal tissue dose constraints with conventional external beam radiation (EBRT). EBRT is often used in combination with IORT to treat a larger elective volume to lower doses, while IORT is used to “boost” the area of greatest concern for local recurrence, or areas that cannot be removed surgically due to their relationship to vital structures or surgical inaccessibility.

Para-aortic lymph node metastases are occasionally encountered in patients with primary or recurrent rectal, colon, cervical, and endometrial cancers, and is a known poor prognostic factor [4–6]. Outcomes of patients with recurrent para-aortic lymph node disease from primary colorectal cancers treated with resection and IORT in recent decades have rarely been reported. As systemic therapies improve and primary therapies allow patients to live longer, the importance of local therapy for low-volume regionally recurrent or metastatic disease increases. We present a retrospective analysis of a series of patients with para-aortic lymph node metastases from recurrent cancer (primarily colorectal) treated with surgical resection and IORT.

## Methods

### Patient characteristics & outcomes

This study was reviewed and approved by an institutional review board. All patients who received IORT were retrospectively identified from 2008 to 2020 at a single institution. Additional inclusion criteria were age ≥ 18 years old, recurrent malignancy in the para-aortic region, and completion of surgical resection with IORT for the recurrence. All patients were included in the reporting of LR and toxicities. Only patients with colorectal primary tumors were included in the overall survival analysis because of the known differences in prognoses of different primary pelvic tumors and few patients with histologies other than colorectal adenocarcinoma.

Patient demographics, oncologic history, and details of IORT and surgical resection were collected from the electronic medical record. This included information related to prior oncologic therapy and details of prior EBRT. Dates of last follow-up, death, and/or progression were used to perform Kaplan Meier analysis for overall survival (OS) and local progression free rate (LPFR). Follow-up, OS, and time to progression were calculated from date of surgery and IORT. Recurrences were considered “in-field” following IORT if the recurrence was in the para-aortic region. We also searched each medical record for toxicities and complications possibly related to the delivery of IORT. These included neuropathy, lymphedema, pulmonary embolism, ureteral obstruction/stricture, renal dysfunction, diarrhea, bowel perforation, bowel obstruction, wound healing delays and dehiscence, and abscess formation.

### Surgical resection & radiation

Patients were initially presented at multidisciplinary tumor boards to confirm the rationale for treatment with surgical resection and IORT. Patients received some combination of surgery, EBRT, and/or chemotherapy as part of definitive management following the initial diagnosis of malignancy. Some patients received preoperative EBRT to the para-aortic region with or without concurrent chemotherapy just before resection and IORT for recurrence. The selected IORT dose was generally not affected by the receipt of preoperative EBRT. All patients underwent surgical resection of the recurrent disease prior to receiving IORT as part of the same procedure. At-risk areas were treated with IORT using an IntraOp-Mobetron (Sunnyvale, CA). Areas were considered at-risk at the discretion of the operating surgeon. Margin status was not available at the time of IORT dose selection. Target volumes were generally void of bowel. Images of the volume treated with IORT were not available. Dose, cone size and shape, and energy for IORT were selected at the time of radiation. IORT doses were prescribed to either the 90% isodose line or the maximum dose and 0.5 cm Lucite bolus was used for all patients.

### Statistical analysis

Data were analyzed using SPSS (IBM, version 27). The significance of association between margin status at time of IORT and LR and pre-operative EBRT and LR was determined using Fisher’s exact test. We performed a Kaplan-Meier analysis of OS (colorectal primary tumors only) and LPFR (all primary tumors) starting follow-up from time of surgery and IORT. For LPFR, death and local-regional recurrence were counted as events. Those lost to follow up were censored for both OS and LPFR analyses.

## Results

### Patient characteristics

The initial search generated a list of 307 patients (all patients receiving IORT at our institution from 2008 to 2020), of which 26 met inclusion criteria, which are listed in the Methods section. The median follow-up was 10.4 months (range: 1.2–96 months) (Table 1). Eight patients received EBRT (all to pelvic volumes) as part of their initial definitive therapy completed a median of 34 months (range: 5–131 months) prior to first recurrence. Two of these 8 patients (both with primary colorectal adenocarcinoma) subsequently received a second course of EBRT for a separate recurrence prior to the recurrence treated with IORT. One of these 2 patients received their second course of EBRT with concurrent chemotherapy for a radiographically identified pelvic recurrence 11 years after definitive therapy. This patient received surgery and IORT for a second recurrence 15 months after the completion of the second course of EBRT. The other of these 2 patients received their second course of EBRT with concurrent chemotherapy 12 months after the completion of initial definitive therapy for radiographically suspicious para-aortic lymphadenopathy with a rising CEA and then went on to receive surgery and IORT 12 months after the completion of the second course of EBRT for recurrent PET-avid para-aortic lymph nodes. No patients had distant metastatic disease at the time of surgery and IORT, aside from the para-aortic lymph node metastases. Additional characteristics are displayed in Table 1.

**Table 1:** Patient characteristics for the entire cohort and only those with colorectal primary tumors. ^*^At time of IORT

	All patients (range)	Patients with Colorectal Primary Tumors (range)
Number of patients	26	19
Median Age (years)^*^	61 (39–79)	59 (39–79)
Sex		
Males	8	7
Females	18	12
Primary Histology		
Colorectal adenocarcinoma	19	N/A
Appendiceal adenocarcinoma	2	
Cervical Squamous Cell	2	
Carcinoma		
Endometrioid Endometrial	1	
Uterine Carcinosarcoma	1	
Ovarian carcinoma	1	
Initial definitive treatment modality		
Surgery	25/26	19/19
EBRT (to pelvis)	8/26	6/19
Chemotherapy	17/26	14/19
Number of patients receiving pre-op EBRT with IORT	11	9
Median follow-up	10.4 (1.2–96.0) months	9.2 (1.2–61.2) months
Median time from initial treatment to recurrence treated with IORT	28 (5–136) months	29 (10–136) months

Eleven separate patients received their first course of EBRT (para-aortic region included in the target volume) as part of treatment for their first recurrence in conjunction with surgery and IORT (median EBRT dose: 45 Gy, range: 32.4–54 Gy, 1.8-2.0 Gy per fraction). Median time from EBRT completion to IORT for these 11 patients was 51.5 days (range: 41–92). Two patients (both with primary colorectal adenocarcinoma) also received IORT to a pelvic site in addition to the para-aortic site.

Fourteen patients were treated with a rectangular IORT field ranging from 12 × 7 cm to 15 × 8 cm (4 of these applicators included a 20 degree bevel). Eleven patients were treated with a circular applicator ranging from 5 to 8 cm in size (3 of these fields included 30–45 degree bevels). IORT applicator size was not available for 1 patient. All patients with positive margins received 15 Gy IORT. For patients with negative margins, the median IORT dose was 15 Gy (range: 10-20 Gy). Additional information is displayed in Table 2.

**Table 2:** Characteristics of IORT for the entire cohort

Characteristic		Number of Patients
Dose	10 Gy	4
	15 Gy	21
	20 Gy	1
Electron Energy	6 MeV	6
	9 MeV	15
	12 MeV	5
Number of Sites Treated with IORT	1	24
	2	1
	3	1

### Recurrence & survival

**Fig. 1:**
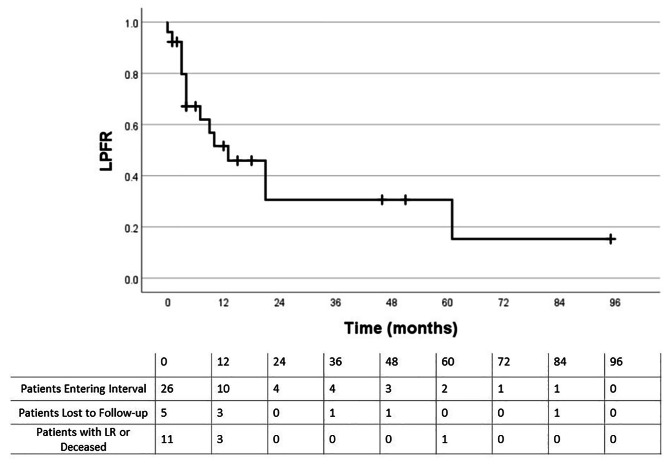
Local progression free rate (LPFR)

**Fig. 2:**
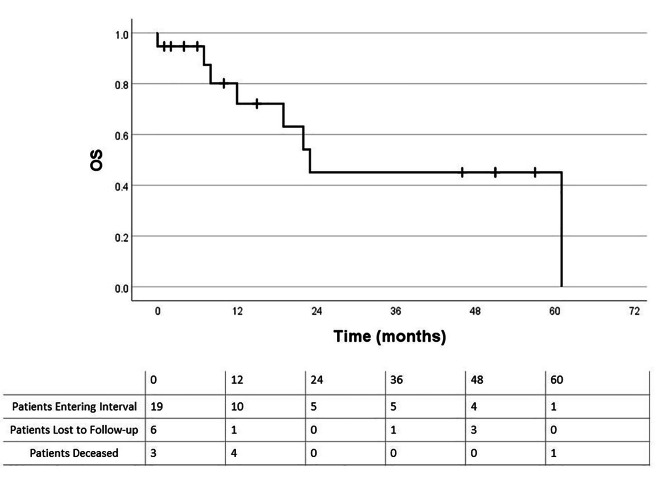
Overall survival for patients with colorectal primary tumors

Recurrences within the IORT field were seen in 31% (8/26) patients. 58% (15/26) of patients developed any cancer recurrence following IORT. Five patients had recurrences in the IORT field only, 7 had out of field recurrences, and 3 had both in-field and out of field recurrences. All 10 out-of-field recurrences were classified as distant metastatic disease. Of the patients with out of field recurrences alone, the median time to recurrence was 12 months (range: 7–33 months). The crude local control (LC) rate in the IORT field was 69% (18/26 patients), which includes patients who received IORT to multiple sites (e.g. para-aortic and pelvic field). Crude LC of para-aortic region only was 77% (20/26 patients). Median time from surgery + IORT to any recurrence was 7 months (range: 1–33 months). For in-field IORT recurrences, the median time from surgery and IORT to subsequent recurrence was 5.5 months (range: 1–10 months). The LR rate was 58% (7/12 patients) for those with positive or close margins vs. 7% (1/14 patients) for those with negative margins (p = 0.009). Of the 12 patients with positive or close margins, 5 had close margins and all were 1 mm or less. All patients that developed local recurrences were treated with either 15 or 20 Gy IORT, rather than 10 Gy. For patients who received pre-operative EBRT vs. those that did not, the LR rate was 36% (4/11 patients) vs. 26% (4/15 patients), respectively (p = 0.683) Table 3 shows recurrence rates for the various subgroups. Kaplan-Meier curves for OS and LPFR are shown in Figs. 1 and 2. Of the deceased, 4 deaths were thought to be due to distant metastatic disease after IORT. The median LPFR was 13 months for the entire cohort. For patients with primary colorectal tumors, the median OS was 23 months.

**Table 3:** Local recurrence rates for various subgroups. “In-Field” refers to the IORT field

Group	In-Field Crude Local Recurrence Rate (%)	p value
Positive or close margins at time of surgery and IORT	58% (7/12 patients)	0.009
Negative margins at time of surgery and IORT	7% (1/14 patients)	
Pre-operative EBRT	36% (4/11 patients)	0.683
No Pre-operative EBRT	27% (4/15 patients)	

### Long-term follow-up (> 4 years)

There were 5 patients in this series with follow-up > 4 years. The OS for this group was 80% at a median of 4.8 years (range 4.3–8 years). Four of these patients had colorectal primary tumors. The remaining patient had a primary endometrial tumor. Two of these patients developed local para-aortic recurrences 7 and 10 months following IORT. The remaining 3 patients developed distant metastatic disease (liver and adrenal metastases) 8, 29, and 33 months following IORT. Three of these patients received chemotherapy following the development of distant metastatic disease and 2 of these 3 patients also received local RFA to the liver.

#### Toxicity

Toxicity data are reported for the entire cohort of 26 patients. 15% (4/26) of patients were diagnosed with surgical wound and/or infectious complications at a median of 19 days (range 10–44 days) following surgery and IORT. These included wound dehiscence, abscess formation, and delayed healing. No patients experienced nerve injury thought to be caused by IORT. One patient developed a foot drop thought to be due to cancer progression along sacral nerve roots identified radiographically. 8% (2/26) of patients experienced lower extremity edema following surgery and IORT. In one of these patients, this was thought to be due to cancer progression in the liver causing hepatic dysfunction. The other patient developed bilateral lower extremity edema immediately following surgery which persisted for 6 months and ultimately required compression devices. One patient was diagnosed with pulmonary embolism during their hospital admission following surgery and IORT.

8% (2/26 patients) of patients experienced diarrhea following IORT. This was due to short gut syndrome following surgery in 1 patient. In another, diarrhea was reported 3 months after IORT. 19% (5/26 patients) of patients developed acute kidney injury (AKI) during their follow-up course after IORT. This was thought to be pre-renal in 3 patients. AKI occurred 1 year later in 1 patient and was thought to be due to poor PO intake. In another patient, AKI was secondary to septic shock which developed the week after IORT. One patient developed an AKI due to the removal of the involved kidney as part of the resection for recurrence and another experienced tumor recurrence causing ureteral obstruction and AKI. There were no reported episodes of bowel perforation or obstruction.

## Discussion

Treatment of recurrent lymph node metastases in the para-aortic region is challenging because local therapies are limited. Surgical resection may leave microscopic- or gross-positive margins and high dose external beam radiation may be too toxic given proximity to nearby sensitive structures (e.g., bowel, kidney). IORT affords the advantage of high-dose irradiation while uninvolved critical structures are physically displaced away from the treatment field. However, there are limited data on efficacy and toxicity with this technique. We report outcomes for patients with para-aortic recurrent malignancy treated with surgery and IORT. The majority of patients in our series had a primary diagnosis of colorectal cancer, with a high risk of local and distant progression.

### Recurrence and survival

Oncologic outcomes for patients treated with surgery and IORT for para-aortic lymph node recurrences have been reported but these were mostly patients with primary gynecologic malignancies with the exception of the reports by Haddock et al. [7–9] Not surprisingly, we report a statistically significant worse LR in patients with positive or close margins compared to negative margins, which has been shown in many cancers but also in recurrent colorectal cancer treated with IORT [10]. IORT may help improve local control in patients known to have a high risk of LR, such as those with gross or microscopic residual disease [9, 11–13]. However, this benefit is difficult to quantify in the absence of randomized data.

Haddock et al. reported a similar series of patients, treated from 1981 to 2000, with colorectal cancer and recurrent para-aortic or mesenteric lymph node disease treated with surgery and IORT. They reported 81% LC at 3 years and 5-year OS of 34%^9^. Calvo et al. reported a LR rate of 14% at median follow up of 39 months and 2-year OS of 57% for patients with a variety of recurrent malignancies treated with surgery and IORT (54% had para-aortic recurrences treated with IORT) [7]. We report patients treated from 2008 to 2020 with similar OS and LC as evidenced by our reported median OS, median time to recurrence, and Kaplan-Meier curves (Figs. 1 and 2). However, significant patient heterogeneity and relatively small number of patients make it difficult to compare results. We acknowledge the limitations of a Kaplan-Meier analysis with few patients available during longer interval follow-up.

Multiple systematic reviews have been published reporting outcomes of surgery without IORT for para-aortic lymph node recurrences in colorectal cancer. Zizzo et al. performed a systemic review for patients with colorectal primary tumors which included 9 studies over the last 30 years. They reported a mean second recurrence rate of 62%, 14–24 months median disease-free survival (DFS), and 3-year OS of 53–88% from the pooled population [14]. 16–29% of re-recurrences were in the para-aortic lymph nodes [14]. Ho et al. also reported results from a systematic review containing 6 larger series for colorectal primary tumors which included a median DFS between 17 and 21 months with a recurrence rate of 67–100% and overall operative morbidity rate of 17–33% [15]. However, many of these recurrences were distant with the exception of those reported by Shibata et al. who reported local recurrences for 11/15 patients who underwent resection with negative margins [12].

The absolute benefit of lymph node dissection and resection for para-aortic recurrences is unclear in this population without prospective randomized data. However, these reviews reported relatively high rates of recurrence and low rates of long-term OS suggesting outcomes may be improved with improved local and systemic therapy. Our in-field recurrence rate of 32% for patients with colorectal primary tumors is similar to previously cited recurrence rates. However, there are many differences including neoadjuvant and adjuvant therapies, comorbidities, cancer burden and biology, and selection bias that make comparisons between studies difficult.

### Toxicity

IORT toxicity has been studied in large retrospective series and a systematic review for patients with primary colorectal cancer [16]. The majority of sites treated with IORT in these cases were in the pelvis. Mirnezami et al. performed a systematic review of locally advanced and locally recurrent rectal cancers treated with surgery +/- IORT. A number of these patients also received EBRT and/or chemotherapy. They found an increased risk of wound complications (delayed healing, wound infection, dehiscence, and “non-specific wound related problems”) in patients who received IORT compared to those who did not (OR 1.86; 95% CI = 1.03–3.38; p = 0.049)^13^. Calvo et al. reported minimal toxicity in patients treated with IORT to para-aortic sites with 1 patient developing a pancreatic fistula who was treated with IORT to the celiac axis [7].

Wound infections and pelvic abscesses have otherwise been reported in approximately 25% of patients but determining the increase in risk due to the addition of IORT to the baseline risk of these complications from surgery is impossible [16]. 15% (4/26 patients) of patients were diagnosed with wound or infectious complications in our study which is similar to previously reported results above. Determining the added side effects of IORT can be difficult in the presence of confounding factors such as surgery and systemic therapy. We felt it was best to report rates of diarrhea and AKI in this group but do not feel that these should all be attributed to IORT.

Studies of IORT in animals have been used to estimate the tolerance of organs and anatomic structures to irradiation. Sindelar et al. studied the effects of IORT to retroperitoneal structures in 14 foxhounds with doses up to 45 Gy [17]. In relation to nerve injury, they reported lower extremity weakness in 1 foxhound approximately 3 years following IORT to 30 Gy [17]. Another study in canines by LeCouteur et al. randomized 85 beagles to receive increasing doses of IORT, EBRT, or both and measured nerve function by electrophysiology or physical exam. They reported a numerical correlation between rates of neuropathy and IORT dose starting at 15 Gy [18]. Notably, nerves within the field were within the 90% isodose line for nearly all subjects in the study [18]. This is not typically true when treating human patients.

Many of our patients experienced peripheral neuropathy due to chemotherapy (oxaliplatin). This and other factors such as nerve-related pain masquerading as musculoskeletal pain may obscure accurate counting of IORT-induced nerve injury. However, no patients in our series suffered from isolated peripheral nerve injury that was thought to be due to IORT. Low rates of neuropathy may also suggest that para-aortic locations for IORT may be favorable as it pertains to IORT-induced peripheral nerve injury which could be due to more accommodating anatomy. The actual radiation dose received by the nerve was also likely much lower than the prescription dose. Thus, we would expect a very low rate of neuropathy due to IORT given these factors and the relatively high-dose tolerance demonstrated by canine studies.

Our study reports a toxicity rate lower than what is typically reported in the literature suggesting IORT to para-aortic sites may cause little or no additional unacceptable toxicity compared to better-studied pelvic sites. One other explanation for the lower toxicity rate compared to other studies in the literature is the number of patients that were followed by their community oncologist after having surgery and IORT at our center. Patients lost to follow-up or followed by physicians outside our health system may have reported complications not recorded in our electronic health record. Surgical techniques and quality have also improved over time which could be responsible for lower toxicity rates compared to studies with patients treated decades ago. Our reported rate of wound complications is in line with previously reported studies of IORT treating non-para-aortic regions [16, 19].

### Limitations

Limitations include the retrospective nature of the study and relatively small cohort. The precise anatomic contents of the IORT field can be difficult to determine based on simple descriptors such as “para-aortic” which can make determining IORT field overlap in patients with prior radiation difficult. The lack of IORT field images makes the discussion of toxicity more difficult. Patients also received EBRT at different times in their disease course (initial definitive management, for a separate recurrence, and preoperatively with surgery and IORT) which can affect recurrence patterns. Many patients in this study were lost to follow-up, often because they were followed at another institution, and this hinders complete collection of oncologic outcomes and toxicities.

## Conclusions

Our study retrospectively evaluates outcomes and toxicity for a group of patients that historically has poor outcomes, as R0 surgical resection is technically difficult in the para aortic region. OS and LR rates are similar to comparable studies with an acceptable toxicity profile using the combination of resection and IORT at our institution. Multidisciplinary discussion is needed to help determine which patients at high risk for recurrence may benefit from the addition of IORT to improve local control.

## Data Availability

The datasets used and/or analyzed during the current study are available from the corresponding author on reasonable request.

## Declarations

### Data sharing Statement

All data generated and analyzed during this study are included in this published article (and its supplementary information files).

## Abbreviations

AKI: Acute kidney injury
DFS: Disease free survival
EBRT: External beam radiation
IORT: Intraoperative electron radiotherapy
LC: Local control
LPFR: Local progression free rate
LR: Local recurrence
PO: Per os
OS: Overall survival
PALN: Para-aortic lymph node
